# Formation of Grown-In Nitrogen Vacancies and Interstitials in Highly Mg-Doped Ammonothermal GaN

**DOI:** 10.3390/ma17051160

**Published:** 2024-03-01

**Authors:** Marcin Zajac, Paweł Kaminski, Roman Kozlowski, Elzbieta Litwin-Staszewska, Ryszard Piotrzkowski, Karolina Grabianska, Robert Kucharski, Rafal Jakiela

**Affiliations:** 1 Institute of High Pressure Physics, Polish Academy of Sciences, Sokolowska 29/37, 01-142 Warsaw, Poland; ela@unipress.waw.pl (E.L.-S.); rp@unipress.waw.pl (R.P.); grabianska@unipress.waw.pl (K.G.); kucharski@unipress.waw.pl (R.K.); 2 Lukasiewicz Research Network—Institute of Microelectronics and Photonics, al. Lotników 32/46, 02-668 Warsaw, Poland; pawel.kaminski@imif.lukasiewicz.gov.pl (P.K.); roman.kozlowski@imif.lukasiewicz.gov.pl (R.K.); 3 Institute of Physics, Polish Academy of Sciences, al. Lotników 32/46, 02-668 Warsaw, Poland; jakiela@ifpan.edu.pl

**Keywords:** GaN:Mg, point defects, charge state changes, ammonothermal growth

## Abstract

The formation of intrinsic point defects in the N-sublattice of semi-insulating Mg-doped GaN crystals grown by the ammonothermal method (SI AT GaN:Mg) was investigated for the first time. The grown-in defects produced by the displacement of nitrogen atoms were experimentally observed as deep traps revealed by the Laplace transform photoinduced transient spectroscopy in the compensated *p*-type crystals with the Mg concentrations of 6 × 10^18^ and 2 × 10^19^ cm^−3^ and resistivities of ~10^11^ Ωcm and ~10^6^ Ωcm, respectively. In both kinds of materials, three closely located traps with activation energies of 430, 450, and 460 meV were revealed. The traps, whose concentrations in the stronger-doped material were found to be significantly higher, are assigned to the (3+/+) and (2+/+) transition levels of nitrogen vacancies as well as to the (2+/+) level of nitrogen split interstitials, respectively. In the material with the lower Mg concentration, a middle-gap trap with the activation energy of 1870 meV was found to be predominant. The results are confirmed and quantitatively described by temperature-dependent Hall effect measurements. The mechanism of nitrogen atom displacement due to the local strain field arising in SI AT GaN:Mg is proposed and the effect of the Mg concentration on the charge compensation is discussed.

## 1. Introduction

Gallium Nitride is now a well-established and commonly used wide-bandgap semiconductor for the fabrication of green, blue, and UV light emitters and high-power electronic devices operating at high voltages above 1 kV [1,2,3]. One of the key issues in the development of GaN-based electronics is the availability of high-quality bulk GaN substrates of controlled conductivity. From the point of view of device architecture, both highly conductive and semi-insulating (SI) substrates are demanded. The former is useful for the production of vertical transistors, the latter is suitable for lateral devices like high-electron-mobility transistors (HEMTs). Precise knowledge of point defect properties in both types of bulk GaN substrates is essential for three reasons: (1) shaping the crystal electrical properties (defect engineering); (2) improving the performance of devices produced by epitaxy on these substrates; (3) thermal behavior of defects (i.e., diffusion) during epitaxy or device operation. 

The ammonothermal (AT) method is one of the key technologies used for growing bulk GaN crystals. It uses supercritical ammonia for the dissolution of feedstock material in order to crystallize bulk GaN on native seeds due to a convection-driven transport [4,5]. The method enables the production of GaN substrates with various conductivity, including *n*-type, *p*-type, as well as semi-insulating (SI) material with a resistivity between 10^6^ and 10^12^ Ωcm. Obtaining the *n*-type conductivity is possible due to the presence of unintentional oxygen (O) donors. The oxygen content, and resulting electron concentration, are of the order of 1 × 10^19^ cm^−3^ and can be reduced by the use of a getter to 1 × 10^18^ cm^−3^. Magnesium (Mg) and Manganese (Mn) are used as dopants playing the role of the acceptor centers compensating the O-related donors in order to obtain AT-GaN crystals with a high resistivity [4]. 

Main native point defects identified both in undoped (*n*-type) and Mg-doped AT-GaN are Ga-vacancy (*V*_Ga_) and *V*_Ga_-related complexes, mainly with hydrogen (*V*_Ga_H_n_, n = 1,2,3) [6,7,8]. *V*_Ga_ is regarded as a compensating acceptor in *n*-type GaN [9]. The estimated concentration of these vacancies in AT-GaN can be of the order of 10^19^ cm^−3^ [6]. The number of hydrogen atoms bound to each vacancy decreases with increasing the electron concentration [7]. It was suggested that in SI-GaN:Mg, the *V*_Ga_-related point defects (mainly *V*_Ga_-H_3_) determine the character of the temperature dependence of material resistivity and carrier concentration [4]. Although the *n*-type conductivity at elevated temperatures and activation energy of *E*_A_ = 1.5 eV, characteristics for the exponential dependence of the resistivity and carrier concentration were measured, the deep defect energy level at 1 eV above valence band maximum (VBM) was speculated to be responsible for this behavior [4]. The location of the (+/0) transition donor energy level (of various *V*_Ga_-related defects) was calculated to be at 1 eV above VBM [10]. It was expected that for the higher Mg concentration with respect to the O content, the Fermi level (*E*_F_) may be pinned to this level resulting in the *p*-type conductivity and *E*_A_ = 1 eV. Indeed, such change in the electrical properties was observed experimentally [11] and these results were interpreted in terms of the Fermi level/compensation-dependent generation of the charge carriers from the deep defect (DD) level located about 1 eV above the VBM and attributed to the *V*_Ga_-related complexes. However, the participation of other intrinsic point defects (apart from *V*_Ga_) in the Mg-acceptors compensation has not been investigated yet, especially as a function of the Mg concentration. In particular, the formation and properties of point defects in N-sublattice in AT-GaN have not been studied. For example, the N-vacancies and their complexes were expected theoretically to be typical compensating donors that inhibit the *p*-type conductivity [12] and this fact was confirmed in GaN:Mg epitaxial layers grown by metalorganic vapor phase epitaxy (MOVPE) [13] and molecular beam epitaxy (MBE) [14]. Their formation energy decreases while the Fermi level approaches the VBM [15,16]. According to first-principle calculations, *V*_N_ is a stable defect in + and 3+ charge states, forming a negative-U center [15]. The transition level corresponding to the transition between + and 3+ charge state (3+/+) was predicted to be located at *E*_V_ + (0.47–0.7) eV, where *E*_V_ is the energy of VBM [15,16,17,18,19,20]. The transitions involving the non-stable 2+ charge states, namely (2+/3+) and (+/2+), were also calculated to be at *E*_V_ + 0.61 eV and *E*_V_ + 0.46 eV, respectively [15]. At the same time, the (0/+) transition level was estimated to be close to the conduction band minimum (CBM) energy. In various works, it was estimated to be at *E*_C_—0.24 eV [19], *E*_C_—0.33 eV [18], and *E*_C_—0.004 eV [21], where *E*_C_ is the energy of CBM.

Attempts to detect nitrogen vacancies (*V*_N_) experimentally were undertaken in many ways. The probing of (0/+) level has not given precise results. From the comparison of Hall-effect and photoluminescence (PL) measurements in electron-irradiated GaN, Look et al. estimated this level to be located at *E*_C_—0.07 eV [22]. The deep-level transient spectroscopy (DLTS) study detected electron trap EE1 at *E*_C_—0.13 eV in *n*-type electron-irradiated GaN [23]. On the contrary, in *p*-type GaN, the hole trap was identified with the level at *E*_V_ + 0.52 eV, but it was not clarified if it had been related to the *V*_N_ (3+/+ state) or nitrogen interstitial (N*_i_*) [23,24]. Usually, the DLTS spectrum is very broad, and the resolution of the observed peaks does not allow for the distinction of different defects of very close energy levels. Nitrogen vacancies are theoretically predicted to be optically active and their deep states were probed by PL. In a highly resistive GaN, they give rise to a green luminescence (GL2) band with a maximum of about 2.35 eV and full width at half maximum (FWHM) of 0.23–0.25 eV [21]. Its shape can be described using a one-dimensional configuration coordinate model diagram with an energy of the zero-phonon line of 2.7 eV, which equals the distance between (+/2+) and (0/+) levels. Moreover, the existence of the excited state of $V_{N}^{+}$ at about 0.1 eV below the CBM (that may correspond to the 0/+ level) must be taken into account in order to explain the exponential decay of PL at T < 20 K and the quenching of the GL2 band with the activation energy of 0.1 eV [21,25]. In addition, at temperatures above 200 K, the quenching of the GL2 band also appears with the activation energy of 0.4–0.65 eV [21]. Thermal emission of holes from the $V_{N}^{+}$ is likely to be responsible for this quenching. Thus, the optical characterization results are in line with the expectations of (0/+) and (+/3+) levels of the *V*_N_ at *E*_C_—(0.1–0.3) eV and *E*_V_ + (0.5–0.7) eV, respectively, from the combination of ab initio calculations, Hall effect data, and DLTS investigation. One should note that the resolution of PL studies to find the position of deep defect levels and determine defect density is not precise, mainly due to the strong electron–phonon coupling and other reasons [26], which makes identification of *V*_N_ centers rather difficult. The most accurate optical studies indicated the position of (+/2+) level at *E*_V_ + (0.7 ± 0.1) eV under the assumption that the excited state is located at *E*_C_ + (0.10 ± 0.03) eV [21,25]. Another technique useful for the detection and characterization of vacancy-related defects in GaN is positron annihilation spectroscopy (PAS). It is very useful for detecting negatively charged Ga vacancies or their complexes. However, this technique has low capability to detect positively charged *V*_N_ centers, especially in bulk crystals [9]. There are only reports identifying the decoration of *V*_N_ centers that form complexes with Mg (Mg_Ga_-*V*_N_) by PAS [13]. Very recently, an individual, isolated *V_N_* on a cleaved m-plane surface of GaN was identified by direct visualization in real space with scanning tunneling microscopy (STM) and atomic force microscopy (AFM) [27]. The thorough and complex identification was established using the results of AFM imaging, analysis of STM images, and tunneling current spectroscopy compared with the results of quantification of the band bending near the surface and first-principles calculations [27]. Nevertheless, the review of the available data published about *V*_N_ in GaN has driven us to the conclusion that despite many theoretical studies indicating the stability of *V*_N_ and showing favorable formation energy of *V*_N_ especially in *p*-type or semi-insulating materials, experimental possibilities to characterize this defect are not sufficient. Therefore, we use Mg doping for the compensation of the residual oxygen shallow donors in the ammonothermal growth of GaN monocrystals. This should move the Fermi level downward, towards the valance band in order to promote the formation of *V*_N_ centers and other accompanying defects related to the nitrogen displacement, like N*_i_* that forms stable complexes in so-called split interstitial (N*_i_*–N*_i_*) configurations [15]. This complex corresponds to two neighboring N atoms sharing the same N site. The energy levels for the charge state transitions of the N*_i_*–N*_i_* defect were calculated theoretically and are located at *E*_V_ + 0.48 eV for the (0/−) transition, and at *E*_V_ + 2.45 eV, *E*_V_ + 0.51 eV, and *E*_V_ + 0.22 eV for the +/0, 2+/+, and 3+/2+ transitions, respectively [15]. They were observed mainly in irradiated samples; for example, by electron paramagnetic resonance (EPR) [28]. We recall that neither *V*_N-_ nor N*_i_*-related defects were observed in GaN crystals grown by the AT method. In this work, we identify deep traps related to N-vacancies and N-interstitials taking part in the compensation mechanism. We demonstrate the first experimental observation of the nitrogen vacancies in addition to the *V*_Ga_ centers in the GaN:Mg ammonothermal crystals. In the high Mg concentration regime, both *V*_Ga_ (0/+) and *V*_N_ (3+/+) may act as deep donors, but the formation energy of the latter should be more favorable. We use the combination of the Hall effect measurements as a function of temperature and the state-of-the-art Laplace transform photoinduced transient spectroscopy (LPITS) to measure the properties and concentrations of deep traps and to identify them with the transition energy levels of *V*_N_ as well as with the transition levels of N*_i_*-related defects. The results are compared for the two kinds of materials: the *p*-type material with the Mg concentration of 2 × 10^19^ cm^−3^ and resistivity of ~10^6^ Ωcm and the highly compensated material, with the Mg concentration of 6 × 10^18^ cm^−3^ and resistivity of ~10^11^ Ωcm.

## 2. Experimental Section

The ammonothermal processes of GaN:Mg were performed at the temperature of 450–550 °C and pressure of 200–400 MPa using a highly reactive supercritical ammonia solution in an appropriate temperature gradient. The dissolved feedstock material, metallic Ga, reacted with the ammonia to soluble species, and Mg of 6N purity was added as the dopant source. The solution was transported via convection to the crystallization zone, where the monocrystalline GaN was deposited on native seeds due to the solution supersaturation. The details of the GaN single crystal growth by the ammonothermal method have been described elsewhere [4,29]. The growth takes place at a high pressure of ammonia, so it can be regarded as a process carried out in the N-rich conditions. The processes aimed at obtaining the GaN single crystals with two different Mg concentrations were performed in the autoclave of 40 mm diameter. In order to accelerate the ammonia reactivity, sodium mineralizer (forming sodium amide at the preliminary growth stage) was introduced into the growth zone. In order to achieve the oxygen concentration of 1 × 10^18^ cm^−3^ in the crystals grown in both processes, an oxygen getter was put into the autoclave. The GaN crystals were grown along the $[000\bar{1]}$-axis and were 1 mm thick and 1 inch or 1.5 inch in diameter, depending on the size of the initial seed. The as-grown crystals were oriented in the c-plane and no post-growth annealing was performed. For the material characterization, the samples from the same slice of the crystal with a thickness of 500 µm were cut out.

The in-depth profiles of Mg, H, O, Zn, Mn, Si, Na, C, and Fe concentrations were determined by the secondary ion mass spectrometry (SIMS), using a CAMECA IMS6F instrument (CAMECA Science & Metrology Solutions, Madison, WI, USA). For the electronegative elements, the measurements were made by means of the cesium (Cs^+^) primary beam, at the energy of 14.5 keV, and a beam current of 200 nA. The size of the raster was about 150 × 150 μm^2^ and the secondary ions H^−^, O^−^, C^−^, and Si^−^ were collected from a central region of a 30-μm diameter. In the case of the electropositive elements, the measurements were performed using the oxygen ($O_{2}^{+}$) primary beam, at the energy of 8 keV and the beam current of 800 nA. The size of the raster was about 150 × 150 μm^2^ and the secondary ions Mg^+^, Zn^+^, Mn^+^, Na^+^, and Fe^+^ were collected from a central region of a 60 μm diameter.

For the characterization by the SIMS and Hall effect measurements, the sample dimensions were 5 × 5 mm^2^. To determine the properties and concentrations of deep traps by the LPITS method, the sample dimensions were 8 × 8 mm^2^. The (0001) surface (Ga-face) of the samples was subjected to mechano-chemical polishing (MCP). The resistivities at room temperature (RT) of the samples labeled #1 and #2 were found to be above 1 × 10^11^ Ωcm and 1 × 10^6^ Ωcm, respectively. The values were determined from the extrapolation to RT and the results were measured in the temperature range of 700–1000 K, in the case of sample #1, and in the range of 400–1000 K, for sample #2. Both kinds of samples were of *p*-type with the Mg concentrations [Mg] by an order of magnitude smaller in the former than in the latter. It is worth adding that sample #1 will be treated as the reference samples in the quantitative analysis which will be presented later in this paper. 

For the Hall effect measurements as a function of temperature, the Ga-face of the 5 × 5 mm^2^ samples was polished mechanically, cleaned, and exposed to inductively coupled plasma (ICP) non-selective etching. On the sample surface prepared in this way, the Ni/Au ohmic contacts in the van der Pauw configuration were deposited and annealed at 500 °C for a few minutes under an atmosphere of N_2_ with a 20% admixture of O_2_. The van der Pauw DC method was used to measure the material resistivity *ρ* and Hall effect in a broad temperature range of 300–1000 K using a set-up consisting of a Keithley 6220 Current Source, Keithley 3706A switch system (Keithley Instruments, Cleveland, OH, USA). For temperature dependent measurements the sample was mounted to a ceramic support and bonded with the Au-wire. The sample holder was installed into the tubular home-built furnace, inserted between electromagnet poles. The applied magnetic field intensity was within the range ±1 T. The temperature was measured and controlled with the Pt resistor.

To perform the measurements of temperature-dependent dark current (TDDC) and mobility-lifetime product (*μτ*) as a function of temperature as well as the measurements aimed at the deep traps characterization by means of the LPITS method, the pairs of ohmic contacts were deposited on the (0001) surface of SI AT-GaN:Mg chips with dimensions of 8 × 8 mm^2^. The two contacts of each pair were separated by a 7 mm gap, providing a window for illumination. The contacts were made by evaporating a 20 nm layer of Cr and a 150 nm layer of Au followed by rapid thermal annealing at 500 °C for 30 s. Finally, each chip was cut into two samples with one pair of electrodes, and a single sample was placed in a thermostatic chamber equipped with a window allowing for illuminating the sample with UV radiation. The temperature of each sample was changed in the range of 300–700 K. All the measurements were made using a Keithley 428 fast current amplifier, a Keithley 2410 (1100 V) SourceMeter (Keithley Instruments, USA), a LakeShore 331 temperature controller (Lake Shore Cryotronics, Westerville, OH, USA), and an Agilent Technologies DSO-X 2022A digital storage oscilloscope (Agilent Technologies, Inc., Santa Clara, CA, USA). The temperature dependences of dark current (TDCC) were carried out at a voltage of 20 V and from the slope of linear parts of the dark current characteristics plotted as log(*T*^3/2^/*I*) versus 1000/*T*, where *T* and *I* denote the absolute temperature and electrical current, respectively, the activation energy of dark conductivity (*E*_ADC_) was determined. The temperature dependences of the *μτ* product for both kinds of samples were determined using the transient photocurrent method (TPM), based on the assumption that *μτ* value at a given temperature is proportional to the amplitude of the photocurrent pulse when the optical pulse generating the excess charge carriers is terminated [30,31]. The *μτ* measurements were carried out at a low flux of 3.31 eV photons incident on the sample, being ~9.7 × 10^14^ s^−1^cm^−2^. The photon pulses produced by the electron-hole pairs were generated by a semiconductor laser emitted by the UV beam with a wavelength of 375 nm. 

The measurements of photocurrent transients were carried out in the temperature range of 300–700 K with an increment of 5 K. The excess charge carriers were generated in the 7 mm gap between the two contacts by a UV beam with a wavelength of 375 nm (3.31 eV) emitted by a semiconductor laser operating in the pulsed mode. The voltage applied between two co-planar contacts was 20 V and the photon flux was 7.9 × 10^17^ cm^−2^ s^−1^. The optical pulse duration time and the pulse repetition period were 10 ms and 500 ms, respectively. The photocurrent transients were amplified and then digitized with a 12-bit amplitude resolution and a 1-μs time resolution. To improve the signal-to-noise ratio, the digital data were averaged by taking 500 waveforms. For further processing, each photocurrent relaxation waveform was normalized with respect to the photocurrent amplitude at the end of the excitation pulse.

## 3. Results and Discussion

### 3.1. Effect of Mg Concentration on SI AT-GaN:Mg Electrical Properties

**Figure 1:**
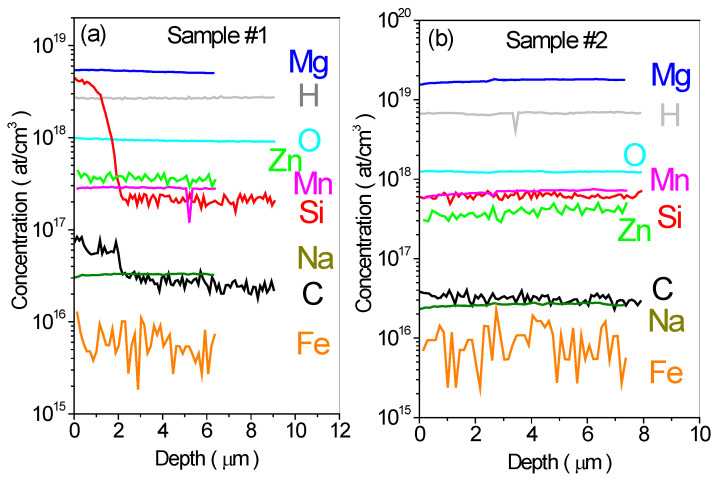
Concentrations of chemical elements detected by SIMS method as a function of depth for two kinds of SI AT-GaN:Mg samples with different Mg doping level. (**a**) Sample #1 with [Mg] = ~6 × 10^18^ cm^−3^. (**b**) Sample #2 with [Mg] = ~2 × 10^19^ cm^−3^.

The results of SIMS measurements, performed on the Ga-face of the samples, are presented in Figure 1. The depth of the crater sputtered by O^−^ and Cs^+^ bombarding ions was up to 8 µm. The depth dependence of the secondary ion count was stabilized after a possible uncontrolled surface contamination due to sample polishing or other surface effects. The in-depth profiles of [Mg], [H], [O], [Zn], [Mn], [Si], [Na], [C], and [Fe] are presented and it is seen that the concentrations of various chemical elements are approximately uniform throughout the whole probed depth. According to the expectations, the predominant chemical element in the two kinds of samples is Mg, whose concentration is 6 × 10^18^ cm^−3^ in sample #1 and 2 × 10^19^ cm^−3^ in sample #2. Because of using a getter, the oxygen concentration is nearly the same in both samples, being at the level of ~1 × 10^18^ cm^−3^. The concentrations of hydrogen in the samples #1 and #2 are 2 × 10^18^ cm^−3^ and 7 × 10^18^ cm^−3^, respectively. This low hydrogen concentration means that if all hydrogen would form Mg-H complexes, only about 30% of Mg would have been passivated. However, the role of hydrogen has not yet been clearly explained yet. It is generally assumed that hydrogen is an amphoteric impurity in GaN, i.e., it can act both as a donor or an acceptor, depending on the Fermi level position [32]. In addition, hydrogen may also form complexes with intrinsic or extrinsic defects. For example, Fourier transform infrared spectroscopy (FTIR) studies of SI AT-GaN:Mg revealed the lines characteristic of the *V*_Ga_H_3_ complex. In the first approximation, we will neglect the influence of hydrogen on the electrical properties of samples #1 and #2 as we assume that the electrically active Mg concentration is high enough to decide the Fermi level position and hydrogen acts only as a small additive to the total donor/acceptor concentration. The concentrations of other residual elements such as Si, Mg, Mn, and Fe do not exceed ~1 × 10^18^ cm^−3^. The background concentrations of these elements (in particular Mn) are too low to affect the electrical parameters of the material. The [Mg], [O], and [H] concentration values and resistivities for the samples used in this study are summarized in Table 1.

The samples whose properties and resistivities are listed in Table 1 represent two kinds of SI AT-GaN:Mg crystals with a different magnesium concentration ([Mg]), the same oxygen concentration ([O]), and a different hydrogen concentration ([H]). It is worth noting that the threefold increase in [Mg] by a factor of 3.3 results in a decrease in the material resistivity by 5 orders of magnitude. According to high-temperature Hall effect measurements, both materials are *p*-type and it can be assumed that the enormous drop in resistivity is due to the huge rise in the hole concentration. In other words, there is an additional reason leading to the very high resistivity of the material with the lower [Mg]. The big difference in the resistivity between the materials with various [Mg] strongly suggests that in the material with lower [Mg], additional deep-level defects are present, which, apart from the Mg_Ga_ acceptors, efficiently compensate the shallow O_N_ donors. These defects are likely to be deep acceptor centers having an energy level near the middle of the bandgap.

**Figure 2:**
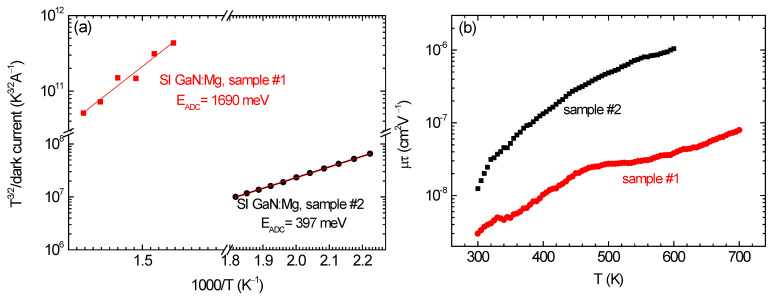
Temperature dependences of the reciprocal of dark current (**a**) and the mobility-lifetime product (**b**) for two SI AT-GaN:Mg samples with [Mg] = 6 × 10^18^ cm^−3^ (sample #1) and [Mg] = 2 × 10^19^ cm^−3^ (sample #2). In both samples [O] = 1 × 10^18^ cm^−3^.

The above-mentioned suggestion is experimentally confirmed by the results of TDDC and mobility-lifetime product (*μτ*) measurements presented in Figure 2, in which the temperature dependences of dark conductivity activation energies (Figure 2a) and the *μτ* product (Figure 2b), established for the two materials with various [Mg], are compared. From a physical point of view, the *E*_ADC_ values given in Figure 2a represent the extrapolated-to-absolute zero Fermi level positions in both kinds of materials [33]. Since the materials are of *p*-type, the Fermi energy values are with respect to the VBM. The results indicate that in the material with the [Mg] = 6 × 10^18^ cm^−3^ (sample #1), the role of magnesium in the charge compensation is very small. This is because the ionized acceptor concentration formed by the electrically active magnesium is too small compared to the ionized donor concentration formed by the electrically active oxygen. In the case of the latter, the ionization level of O_N_ is at *E*_c_—33 meV and at 300 K all the oxygen atoms, whose concentration is 1 × 10^18^ cm^−3^, are positively ionized [34]. In the case of magnesium, the ionization process is more complex, since a part of the [Mg] is passivated by hydrogen. Assuming that 30% of the [H] given in Table 1 contributes to the Mg passivation, which equals 9 × 10^17^ cm^−3^, the Mg concentration that can be electrically active is 5.1 × 10^18^ cm^−3^. Moreover, the Mg_Ga_ ionization level is relatively deeply located in the bandgap, at around *E*_v_ + 200 meV, and at RT typically only a few percent of the non-passivated Mg_Ga_ is negatively ionized [34]. Taking that this part is 10% [34], the concentration of the negatively ionized acceptors Mg_Ga_^−^ in the material with the total [Mg] = 6 × 10^18^ cm^−3^ (sample #1) is 5.1 × 10^17^ cm^−3^. Thus, the O_N_ shallow donors in this material are predominantly compensated by deep acceptors having the ionization level located closely to the Fermi level position given by the *E*_ADC_ = *E*_v_ + 1690 meV.

In the material with the [Mg] = 2 × 10^19^ cm^−3^ (sample #2), the compensation mechanism is entirely different. This fact is indicated by the Fermi level position given by the *E*_ADC_ = *E*_v_ + 397 meV, which is much closer to the VBM. The significant shift of the Fermi level towards the VBM with increasing [Mg] seems to be due to three reasons. Firstly, the compensation is not affected by the very deep acceptors with the ionization level located in the middle of the bandgap. Secondly, the concentration of negatively ionized acceptors ${Mg}_{Ga}^{-}$ exceeds the concentration of positively ionized shallow donors $O_{N}^{+}$. This fact can be confirmed by a rough calculation, taking into account that the [H] = 7 × 10^18^ cm^−3^ (see Table 1), and assuming that 30% of the [H] takes part in the Mg passivation. In this way, the concentration of the non-passivated Mg that can be electrically active is 1.79 × 10^19^ cm^−3^, and taking into account that only 10% of these Mg atoms are ionized, the concentration of negatively ionized acceptors ${Mg}_{Ga}^{-}$ is 1.79 × 10^18^ cm^−3^. Thirdly, the Fermi level in the material (sample #2) is located clearly above the Mg_Ga_ level and this fact indicates that other acceptors, with deeper ionization levels, are likely to be also involved in the compensation of the $O_{N}^{+}$. shallow donors. 

The temperature dependences of the *μτ* product presented in Figure 2b are consistent with the Fermi energies given in Figure 2a for both kinds of materials with the various [Mg]. It is seen that increasing the [Mg] from 6 × 10^18^ cm^−3^ (sample #1) to 2 × 10^19^ cm^−3^ (sample #2) and keeping the [O] = 1 × 10^18^ cm^−3^ leads to a substantial rise in the *μτ* values determined in the temperature range of 300–700 K. However, for the latter material, the measurements were stopped at 600 K due to a high dark current. It is worth noting that at 300 K, the ratio of the *μτ* values for samples #2 and #1 is around 4, but at 600 K, this ratio is around 20. In other words, the temperature increase in the *μτ* is stronger for sample #2 than for sample #1. Since the *μτ* product determines the quality of semiconductor material in terms of its application for the fabrication of various kinds of sensors, used as photodetectors, X-ray detectors, and radiation detectors, the results shown in Figure 2b are of practical importance [31,35]. They demonstrate that by increasing the doping level in SI AT-GaN:Mg crystals, it is possible to obtain the material suitable for making unique detectors operating at 600 K. In view of the fact that above 300 K the mobility of charge carriers decreases with temperature due to the lattice scattering, the observed in Figure 2b rise in the *μτ* values as a function of temperature can only result from the strong increase in the excess charge carriers lifetime [35]. The lifetime strongly depends on the properties and concentrations of deep-level defects acting as the recombination centers and the ionization levels of the most efficient recombination centers are located in the vicinity of the middle of the bandgap [35]. According to the results shown in Figure 2a, the efficient recombination centers are likely to be present only in the material with the lower [Mg] (sample #1). The observed changes in the lifetime against temperature reflect the changes in the charge carrier recombination rate, being equal to the lifetime reciprocal. In other words, for both kinds of materials, the recombination rate decreases with temperature, and in the case of deeper recombination centers (sample #1), the decrease is slower than for the centers whose ionization levels are located closer to the VBM or CBM [35]. In view of the SRH recombination model, proposed by Shockley, Read, and Hall [36,37], the recombination takes place when an electron from the conduction band is captured by the empty defect level and then a hole from the valence band is captured by an electron occupying this level. With increasing temperature, the thermal emission rate of charge carriers rises exponentially and the probability of releasing the electron or hole from a defect center to the conduction or valence band becomes much higher than that of their capture by this defect center [35].

### 3.2. Effect of Mg Concentration on the Properties and Concentrations of Deep Traps

In the LPITS method, the occupation of defect levels is changed by the capture of excess charge carriers excited by optical pulses and after the excitation is switched off, the photocurrent relaxation waveforms (PRWs) induced by the thermal emission of excess electrons or holes from the defect levels are produced [31]. At a given temperature, the PRW is observed as a bottom part of the photocurrent decay, occurring in the photocurrent transient when the optical excitation is switched off. Usually, the PRW represents the slower part of the photocurrent decay that appears for times longer than ~1 μs from the termination of the optical pulse. The faster decay rate, which is observed for the shorter times, is related to the excess charge carrier recombination. Thus, the PRWs are the distinctive parts of the photocurrent transients and their analysis as a function of temperature enables the properties and concentrations of charge carrier traps to be determined [31]. Most frequently, a PRW observed in the photocurrent transient recorded at a given temperature is composed of a few exponential signals related to the thermal emission of charge carriers, and an advanced numerical procedure is used to extract the time constant of each exponential signal [31]. This procedure, based on the inverse Laplace transformation algorithm (ILT), uses the CONTIN code [38] adapted to the commercially available MATLAB computational environment. Assuming that retrapping excess charge carriers is neglected, the time dependence of a PRW exponential component at a given temperature *T* can be written as [31]

*I*(*t*) = *I*(0) exp(−*e*_*T*_*t*), (1)

where *t* denotes time, *e*_*T*_ is the charge carrier thermal emission rate characteristic of the defect center that captured the carriers when the sample was illuminated, and *I*(0) is the amplitude of the exponential signal when the optical excitation pulse is terminated. The *e*_*T*_ is equal to the reciprocal of the exponential signal time constant and the *I*(0) can be expressed in the form [31]

*I*(0) = *qn*_*T*_ (0)*e*_*T*_*µτEC*, (2)

where *n*_*T*_ (0) is the concentration of electrons or holes trapped by the defect center at *t* = 0 when the optical excitation pulse is switched off, *q* is the elementary charge, *E* is the electric field between two planar contacts dependent on the applied voltage and *C* is the geometrical parameter equal to the area of the cross-section of a sample region through which the excess charge carriers emitted from the defect center flow to the electrodes on the sample surface. The temperature dependence of the excess charge carrier thermal emission rate is given by the Arrhenius equation [31]

*e_T_*(*T*) = *AT*^2^exp(−*E*_a_/*k*_B_*T*), (3)

where *E*_a_ is the activation energy, *k*_B_ is the Boltzmann constant, *A* = *γσ*_a_ is the pre-exponential factor, equal to the product of the material constant γ, dependent on the effective mass, and the apparent capture cross-section for electrons or holes *σ*_a_. Thus, each defect center is characterized by the activation energy *E*_a_ for electron or hole thermal emission and pre-exponential factor *A*. For GaN with wurtzite structure, the values of *γ*_n_ and *γ*_p_, necessary for obtaining the apparent capture cross-sections for electrons or holes, are 6.48 × 10^20^ and 6.33 × 10^21^ K^−2^ cm^−2^ s ^−1^, respectively.

To extract the parameters *E*_a_ and *A*, characterizing defect center properties, the PRWs recorded in the time domain at temperatures *T*_j_ (j = 1, 2, 3, ……) within the range of 300–700 K, were transformed into the one-dimensional (1D) Laplace spectra *S*_Lj_ in the domain of the thermal emission rate in which the sharp peaks indicated the *e_T_* values characteristic of the defect centers detected at each temperature. Next, all the 1D Laplace spectra were assembled to create the (2D) Laplace spectrum which visualized in the 3D space contained the sharp folds whose ridgelines depicted the temperature dependences of the emission rate for the defect centers that had trapped the charge carriers during the sample illumination [31]. The *T* and *e_T_* data derived from these ridgelines were used to draw the Arrhenius plots in coordinates ln(*T*^2^/*e_T_*) = f(1/*k_B_T*), being the signatures of defect centers. For each trap, the activation energy *E*_a_ and the pre-exponential factor A in the Arrhenius equation were determined from the slope and intercept of the Arrhenius plot by means of linear regression. The concentration (*N*_T_) of a defect center was determined from the amplitude of the exponential component of the PRW observed at a given temperature. According to Equation (2), the amplitude *I*(0) is proportional to the concentration *n_T_*(0) of charge carriers trapped by the defect center when the optical excitation pulse is switched off. To find the experimental values of *I*(0) for the exponential signals resulting from the analysis of the PRW measured at a given temperature, the waveform was fitted with the sum of exponential signals in the form of Equation (1), whose number and time constants were derived from the 1D Laplace spectrum. More details on extracting the trap concentration from LPITS measurements is given elsewhere [31]. 

**Figure 3:**
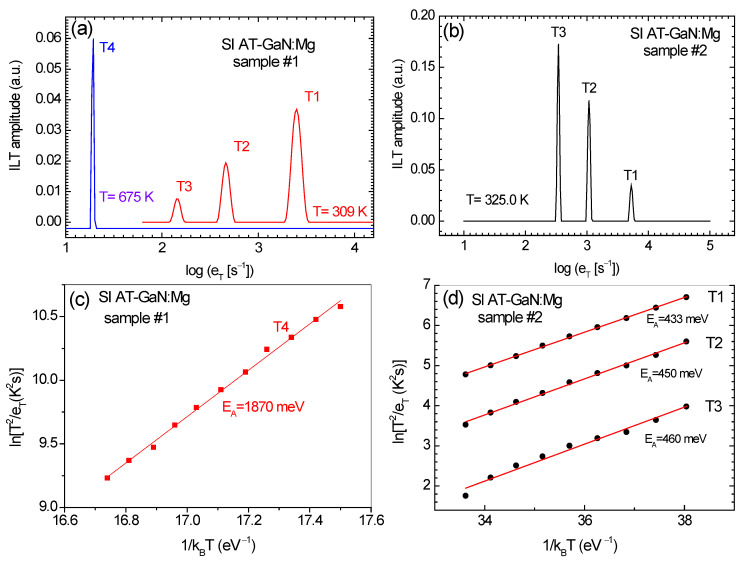
(**a**) One-dimensional Laplace spectra derived from the photocurrent relaxation waveforms recorded at 309 K (in red) and 675 K (in blue) for a sample of SI AT-GaN:Mg crystal with [Mg] = 6 × 10^18^ cm^−3^. (**b**) One-dimensional Laplace spectrum derived from the photocurrent relaxation waveform recorded at 325 K for a sample of SI AT-GaN:Mg crystal with [Mg] = 2 × 10^19^ cm^−3^. (**c**) Arrhenius plot for the thermal emission of charge carriers from the T4 trap detected only in a sample of SI AT-GaN:Mg crystal with [Mg] = 6 × 10^18^ cm^−3^. (**d**) Arrhenius plots for the thermal emission of charge carriers from the T1, T2, and T3 traps detected in samples of SI AT-GaN:Mg crystals with [Mg] = 6 × 10^18^ cm^−3^ and [Mg] = 2 × 10^19^ cm^−3^.

The characteristics of charge carrier traps determined by the LPITS for two kinds of SI AT-GaN:Mg crystals with the [Mg] = 6 × 10^18^ cm^−3^ (sample #1) and [Mg] = 2 × 10^19^ cm^−3^ are shown in Figure 3. The results were obtained by applying the CONTIN procedure to the analysis of the sets of the photocurrent transients generated in the samples of these crystals at temperatures 300–700 K. Figure 3a,b shows the one-dimensional Laplace spectra indicating the presence of three exponential components related to the thermal emission of charge carriers in the photocurrent relaxation waveforms observed for the samples #1 and #2 at temperatures 309 and 325 K, respectively. In addition, Figure 3a contains the one-dimensional Laplace spectrum indicating the thermal emission of charge carriers from deep-level defects at 675 K. It is worth noting that the three traps, which manifest themselves through the Laplace peaks labeled as T1, T2, and T3, are present in both kinds of crystals. The fourth trap, observed at 675 K through the Laplace peak labeled as T4, is only detected in the material with the [Mg] = 6 × 10^18^ cm^−3^. 

According to the data shown in Figure 3a,b, the thermal emission rates at 309 K for the T1, T2, and T3 traps are 2512, 479, and 145 s^−1^, respectively, and at 325 K are 5129, 1072, and 339 s^−1^, respectively. For trap T4, present only in the material with the lower [Mg], the thermal emission rate of charge carriers at 675 K is 19 s^−1^. The dependence of the thermal emission rate reciprocal as a function of the thermal energy reciprocal for this trap is shown in Figure 3c. The dependences of the thermal emission rate reciprocals plotted against the thermal energy reciprocal for the T1, T2, and T3 traps are shown in Figure 3d. 

Each Arrhenius plot is the trap signature and is used to determine the parameters of the Arrhenius equation characterizing the temperature dependences of the thermal emission rate of electrons or holes. The slope of the plot allows finding the activation energy *E*_a_ for the thermal emission of charge carriers from the trap and the intercept gives the pre-exponential factor A in the Arrhenius equation. The *E*_a_ value for the T4 trap is given in Figure 3c and the *E*_a_ values for the T1, T2, and T3 traps are shown in Figure 3d. All the deep trap parameters extracted from the Arrhenius plots presented in Figure 3c,d as well as the trap concentrations estimated from the amplitudes of the exponential signals found in the photocurrent relaxation waveforms recorded for both kinds of samples of SI AT-GaN:Mg crystals are listed in Table 2.

The LPITS results summarized in Table 2 reveal the qualitative and quantitative changes in the defect structure of SI AT-GaN:Mg crystals induced by increasing the [Mg] from 6 × 10^18^ to 2 × 10^19^ cm^−3^. The qualitative result is that the T4 trap is present only in the material with the lower [Mg]. Moreover, the activation energy value of this trap is close to the Fermi energy value of *E*_v_ + 1690 meV derived from the TDDC measurements. Quantitatively, the results in Table 2 indicate that the T4 trap predominates in the material with the lower [Mg] and its concentration is near that of the T1 trap in the material with the higher [Mg] in which this trap is predominant. On the other hand, the T1 trap concentration in the former is the lowest compared to the concentrations of traps T2 and T3. It is worth noting that the T2 and T3 trap concentrations in the latter material are by the order of magnitude higher than that in the former, but in both materials, the inequality [T2] > [T3] is fulfilled. Moreover, a fact worth emphasizing is that the activation energy of the T1 trap, which is predominant in the material with the higher [Mg], is close to the Fermi energy value of *E*_v_ + 397 meV obtained for the material by the TDDC measurements. It should be added that the LPITS measurements do not allow us to distinguish directly between the electron or hole traps. However, by using both parameters *E*_a_ and *A*, the properties of traps detected by LPITS with those found by DLTS can be indirectly compared.

The parameters determined from the Arrhenius plot for the T1 trap well match those for the hole trap DP1, with the activation energy and pre-exponential factor of *E*_v_ + 484 meV and 1.77 × 10^4^ K^−2^s^−1^, respectively, detected by the current transient method in the *p*-type GaN film playing role a gate in a high-electron-mobility transistor (HEMT) [39]. This trap, similarly to the other hole traps found in a *p*-GaN HEMT and *p*-GaN Schottky diodes with the *E*_a_ of 480–490 meV and *A* in the range of 10^3^–10^4^ K^−2^s^−1^, was suggested to be attributed to nitrogen vacancies [40]. A trap with parameters close to those of the T3 trap is the hole trap EHa revealed by DLTS in homoepitaxial *p*-type GaN with the Mg concentration of 8 × 10^15^ cm^−3^ after the irradiation with 137-keV electrons to produce nitrogen atoms displacement [24]. The *p*^+^/*p*^−^/*n*^+^ GaN epitaxial structures exposed to the electron bombardment were grown by metalorganic vapor phase epitaxy (MOVPE) on freestanding GaN substrates. The activation energy and pre-exponential factor derived from the Arrhenius plot for the EHa trap are *E*_v_ + 520 meV and 1.9 × 10^6^ K^−2^s^−1^, respectively [24]. On the grounds of the first-principle calculations based on a hybrid functional, this trap was proposed to be attributed to *V*_N_ (3+/+) or N*_i_*(2+/+). It is worth adding that with increasing the electron fluence from 4.6 × 10^15^ to 1.9 × 10^16^ cm^−2^, the EHa trap concentration went up from 4.0 × 10^14^ to 2.3 × 10^15^ cm^−3^, respectively [24]. In turn, the T2 trap parameter values are comparable with the *E*_a_ = *E*_v_ + 480 meV and *A* = 2.3 × 10^5^ K^−2^s^−1^ determined by DLTS for a Mg-doped *p*-type GaN epitaxial layer exposed to the irradiation with 1.8-MeV protons [41]. The layer with a [Mg] = 2 × 10^17^ cm^−3^ was grown by ammonia-based molecular beam epitaxy [41]. Before the irradiation, the *E*_v_ + 480-meV trap concentration in the material was ~10^13^ cm^−3^, and after irradiations with proton fluences of 1 × 10^13^ and 3 × 10^13^ cm^−2^, its concentration increased to 3.8 × 10^14^ and 6.4 × 10^14^ cm^−3^, respectively [41]. Finally, the T4 trap with parameters listed in Table 2 is likely to be related to the same defect as the hole trap H5 detected by minority carrier transient spectroscopy (MCTS) in samples of *n*-type GaN grown by MOCVD on free-standing *n*^+^-GaN substrates [42]. The values of the *E*_a_ and *A* parameters for this trap derived from the Arrhenius plot are *E*_v_ + 1760 meV and 7.6 × 10^9^ K^−2^s^−1^, respectively [42]. The lower activation energy for the hole thermal emission from the trap H5 and higher pre-exponential factor *A*, proportional to the hole capture cross-section, can result from the substantially higher electric field strength under the MCTS experiment than that under the LPITS measurements [42]. In the case of the former, the trap was probed in the space charge region of a *p*^+^-*n* junction where the electric field is of the order of 10^6^ V/cm. In the case of the latter, the hole thermal emission from the trap T4 was induced in the homogeneous SI GaN sample at the electric field of ~286 V/cm. It is worth adding that the red PL bands, with peak positions around 1.8 eV, have been observed in both *p*- and *n*-type GaN, but their origins remain unknown [19,43]. 

### 3.3. Identification of Detected Traps Based on HSE-Hybrid Functional Calculations

The comparison of the T1–T4 trap parameters given in Table 2 with those of derived from the Arrhenius plots determined by other methods used to observe and analyze the thermal emission of charge carriers in *p*-type GaN allows us to conclude that the traps detected by LPITS are hole traps and their activation energies refer to the VBM. The next step in their identification is to propose the atomic configurations, as well as the charge state changes associated with the thermal emission of holes, and to establish whether they are donors or acceptors. All these facts can be found from the results of simulating the formation energy and properties of native defects in GaN obtained by the ab initio calculations made using the Heyd, Scusseria, and Ernzherof (HSE) range-separated hybrid functional as well as the projector-augmented wave (PAW) formalism implemented in the Vienna Ab-initio Simulation Package (VASP) code [15,16]. The recent results of these calculations are sufficiently unambiguous to be used for the comparison of the activation energies of experimentally detected deep traps with the known transition levels of native defects in GaN. The electronic properties of native point defects identified in both kinds of samples of SI AT-GaN:Mg crystals on the grounds of the experimental results obtained by LPITS measurements are summarized in Table 3. The comparison given in Table 3 indicates that the experimentally determined activation energies for hole emission from the T1, T2, and T3 defect states related to the nitrogen displacement from the substitutional sites are within 40 meV lower than the energies calculated by using the HSE hybrid functional corresponding to the defects charge state transitions. This fact can be accounted for by assuming that the defects, whose activation energies with an accuracy of about ±10 meV are derived from the LPITS measurements, are influenced by a deformation potential induced by the local strain introduced into the lattice of SI AT-GaN:Mg crystals due to the size mismatch between Mg and Ga atoms [44]. The atomic radii of Mg and Ga are 140 and 123 pm, respectively, and the large difference between them results in two effects: a size effect, related to the lattice deformation and an electronic effect, related to the creation of the deformation potential [44,45]. As far as the size effect is concerned, the calculations performed by Van de Walle [44] indicate that the nitrogen atoms in the first coordination sphere surrounding the ${Mg}_{Ga}^{0}$ relax outwards by 6.1% of the bulk Ga-N bond length in the direction parallel to the *c* axis. On the other hand, in the second coordination sphere, the nitrogen atoms are compressed due to a small atomic radius (71 pm) and can be easily removed from the substitutional to interstitial positions by the elastic force [44,45]. In other words, a change in the strain state from compressive to tensile is likely to be accompanied by the generation of nitrogen interstitials and nitrogen vacancies [43,44]. There are also two experimental facts confirming this change [43]. The first is obtained by Raman spectroscopy and shows that the strain-sensitive E_2_(high) mode of the Raman shift is monotonically reduced for Mg-concentrations higher than 7 × 10^18^ cm^−3^ [43]. With increasing the [Mg] in the material, the compressive strain goes down, and for [Mg] as high as 2 × 10^19^ cm^−3^ no symmetry-forbidden modes are observed [43]. The second is derived from the photoluminescence measurements indicating that the change in strain state is correlated with the change in optical spectra involving a saturation of the DAP luminescence and enhancing the signals related to the transitions induced by deep-level defects [43]. The results allow us to conclude that the change in the strain state is accompanied by the change in the optical electron transitions, for the predominant contribution of the shallow states to the photon emission is replaced by the contribution of deep states [43]. 

The T1 trap activation energy of 433 meV compares well with the *V*_N_ (3+/+) donor level, which according to the HSE calculations made by Lyons and Van de Walle [16], is located at 450 meV above the VBM. It is worth adding that by means of HSE calculations with finite-size corrections, Yan et al. found the *V*_N_ (3+/+) level position at 470 meV above the VBM [19]. In turn, the HSE calculations performed by Diallo and Demchenko [15] gave the *V*_N_ (3+/+) level position at 540 meV above the VBM. These data illustrate that there is a spread in the values of the HSE-calculated transition energies. The discrepancies in the calculated transition levels can be due to the differences in the lattice relaxations of a defect, various supercells sizes, and the **k**-point sampling methods, as well as due to the use of different electrostatic correction scheme [15]. The possibility of the (3+/+) charge state change for the nitrogen vacancy is predicted by most HSE calculations [15,16]. This means that *V*_N_ exhibits the properties of a negative-*U* center that being singly positively ionized needs to capture two holes to be transferred to the (3+) charge state and the binding energy of these holes is greater than in the case of the one-hole capture [15,16]. The experimental fact that the T1 trap concentration in the material with the [Mg] = 2 × 10^19^ cm^−3^ (Table 2) is two times higher than that of the T2 trap, identified with the *V*_N_ (2+/+), supports assigning to the T1 trap the negative-U defect properties. Attributing the T1 trap to *V*_N_ (3+/+) means that during the sample illumination, this trap captures two holes and the charge released by the thermal emission when the illumination is switched off is two times larger than in the case of the T2 trap [31]. Actually, both trap concentrations are the same; however, the amplitude of the exponential component of the photocurrent relaxation waveform, which is used to determine the trap concentrations, is doubled in the case of the thermal emission from the T1 trap [31]. The values of the capture cross-section for holes (Table 3) are also in line with the proposed identifications of T1 and T2 traps. For the former, the *σ*_p_ value is nearly by the order of magnitude lower due to the stronger Coulomb repulsive force while capturing two holes simultaneously. 

The trap T3 is proposed to be attributed to the N split interstitial N*_i_*–N*_i_*. The trap activation energy of 460 meV is close to the donor level related to the (2+/+) charge state change in this defect whose location in the bandgap calculated by Kyrtsos et al. is at *E*_v_ + 500 meV [17] and by Diallo and Demchenko at *E*_v_ + 510 meV [15]. It should be noted that the activation energy for the hole emission is likely to be diminished due to the deformation potential in the defect’s vicinity [15,44]. In the calculations, the initial N*_i_*–N*_i_* bond length is assumed to be 0.113 nm, which well matches the bond length of a free N_2_ molecule (0.11 nm) [15]. The HSE calculations show that the N split interstitial can capture one electron, becoming singly negatively ionized, or up to three holes, being in the (+), (2+), and (3+) charge states [15]. When illuminating the SI AT-GaN:Mg sample during the LPITS experiment, the N*_i_*–N*_i_* (+) captures an excess hole and goes to the (2+) charge state. After switching off the optical excitation, the hole is thermally released to the valence band and the defect returns to the initial charge state. It is worth adding that the N*_i_*–N*_i_* bond distance is dependent on the charge state and for the extreme cases (−) and (3+) are 0.141 and 0.111 nm, respectively [15]. 

The results listed in Table 3 indicate that during the growth of SI AT-GaN:Mg crystals in N-rich conditions, the formation of nitrogen vacancies is correlated with the formation of nitrogen interstitials. In other words, the compressive strain induced by the large Mg atomic radius (0.140 nm) results in the formation of Frenkel pairs, which are two separated defects: a nitrogen vacancy and a nitrogen interstitial. Because of the small nitrogen atomic radius (0.071 nm), the latter is very mobile at the growth temperature of ~500 °C [46]. Using density-functional total energy calculations, Wixom and Wright [46] have shown that the N interstitial migration is very effective both perpendicular and parallel to the *c*-axis, since there are low barriers for the defect migration in the all-positive charge states: (+), (2+), and (3+). According to the calculated results, the N interstitial diffusion length strongly increases with temperature and at ~500 °C is of the order of 1000 nm. On the other hand, the results shown in Table 2 and Table 3 indicate that in the SI AT-GaN:Mg samples with the Mg concentrations of 6 × 10^18^ and 2 × 10^19^ cm^−3^ the concentrations of N interstitials are 3.6 × 10^17^ and 3.6 × 10^18^ cm^−3^, respectively. These values allow us to conclude that the average distance between the defects in the materials with the lower and higher Mg doping level are 14 and 6.5 nm, respectively. Thus, the probability of the N*_i_*–N*_i_* split interstitials formation during the growth of SI AT-GaN:Mg crystals is very high. It should be stressed that for the material of each kind the [N*_i_*] and [*V*_N_] values are comparable and this fact supports the view that the Frenkel pairs are created during the growth of SI AT-GaN:Mg crystals. 

To characterize the impact of the impurity concentration on the size effect, the coefficient *β*size which relates the fractional change in the lattice parameter (Δ*a*/*a*) to the impurity concentration was calculated [44]. Thus, for Mg-doped GaN, the Δ*a*/*a* = *β*size × [Mg_Ga_], where the *β*size = 1.5 × 10^−24^ cm^3^ [44]. Consequently, for the Mg concentrations in the SI AT-GaN:Mg, crystals equal to 6 × 10^18^ and 2 × 10^19^ cm^−3^, the Δ*a*/*a* values are 9 × 10^−6^ and 3 × 10^−5^, respectively. These positive changes in the lattice parameter should be compensated by the negative changes induced by the formation of N vacancies in the N-sublattice [24,43]. The -Δ*a*/*a* can be defined as [*V*_N_]/[N_N_], where [*V*_N_] is the nitrogen-vacancy concentration and the [N_N_] is the concentration of nitrogen atoms in the N-sublattice which is known to be equal to 4.45 × 10^22^ cm^−3^. In the materials with the Mg concentrations of 6 × 10^18^ and 2 × 10^19^ cm^−3^, the N vacancy concentrations determined from the LPITS measurements are 2.8 × 10^17^ and 4.0 × 10^18^ cm^−3^, respectively, which gives the −Δ*a*/*a* values of 6.3 × 10^−6^ and 9.0 × 10^−5^, respectively. The fact that the results obtained by the theoretical calculations and by the LPITS studies are of the same order confirms the strain involvement in the formation of the Frenkel pairs. 

The T4 trap activation energy of 1870 meV exactly matches the HSE-calculated acceptor level of gallium vacancy (*V*_Ga_) located at *E*_v_ + 1870 meV [15]. The level is related to the hole thermal emission from the *V*_Ga_ (−), resulting in the (−/2−) charge state transition. When the sample is illuminated during the LPITS measurements, the *V*_Ga_ (2−) captures an excess hole from the valence band, becoming *V*_Ga_ (−), and after switching off the illumination, the hole is thermally emitted back to the valence band. In other words, in the equilibrium conditions, the *V*_Ga_ is doubly negatively ionized and effectively compensates the singly positively ionized oxygen-related shallow donors. This is the case for the material with the [Mg] = 6 × 10^18^ cm^−3^ in which the *V*_Ga_ (−/2−) is experimentally detected through the T4 trap whose concentration is found to be ~1 × 10^19^ cm^−3^. It should be emphasized that this trap has not been detected in the material with the [Mg] = 2 × 10^19^ cm^−3^. This result is of great importance since it indicates that under the N-rich conditions during the crystal growth, the *V*_Ga_ are formed as non-stoichiometric defects due to the presence of an excess of nitrogen [47]. A similar phenomenon occurs under the growth of undoped GaAs crystals when *V*_Ga_ are formed due to a small deviation from stoichiometry under the As-rich conditions [48]. In other words, Mg-doping consumes the Ga vacancies present in the material by the reaction Mg*_i_* + *V*_Ga_ → Mg_Ga_. Therefore, it can be assumed that in the material with the [Mg] = 6 × 10^18^ cm^−3^, the initial [*V*_Ga_] had been 1.6 × 10^19^ cm^−3^, and during the crystal growth the part of this concentration was filled with the Mg interstitials. On the other hand, in the material with the [Mg] = 2 × 10^19^ cm^−3^, the initial [*V*_Ga_] had been presumably the same; however, all the Ga vacancies were filled with the Mg interstitials, for the [Mg] is higher than [*V*_Ga_]. Thus, the T4 trap, identified with the *V*_Ga_ (−/2−), has not been detected in this material by the LPITS measurements. The proposed interaction of Mg with *V*_Ga_ is consistent with the positron annihilation spectroscopy results obtained by the studies of grown-in vacancy defects in bulk GaN crystals grown by the ammonothermal method [6]. A high concentration of *V*_Ga_-related defects was observed only in the undoped with Mg *n*-type samples. In the Mg-doped samples, with the [Mg] of the order of 10^19^ cm^−3^, no positron trapping at vacancy defects was observed [6]. 

### 3.4. Compensation Mechanism 

**Figure 4:**
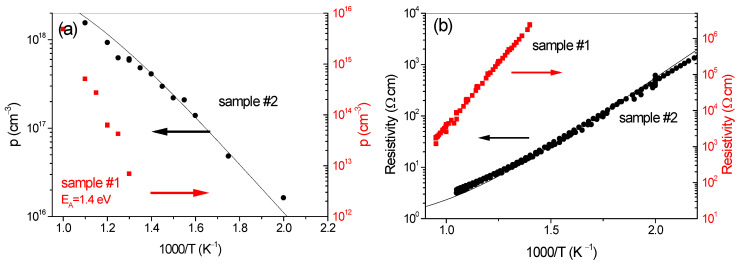
The temperature dependence (**a**) of hole concentration, (**b**) of resistivity for samples #1 (red, right axis) and #2 (black, left axis). The solid black lines represent the solution of the charge neutrality equation to results of sample #2 with the following parameters: *N_Mg_* = 2 × 10^19^ cm^−3^, *N_DO_* = 1 × 10^18^ cm^−3^, *N_DD_* = 1.1 × 10^19^ cm^−3^, *E_Mg_* = 150 meV (*g_Mg_* = 4) above VBM, *E_DD_* = 0.45 meV (*g_DD_* = 1) above VBM. A linear temperature dependence of the *E_DD_* was assumed (*E_DD_ = E_DD_(T = 0)* + *γT)* with proportionality factor *γ* = −0.35 meV/K, which means that both CBM and VBM approach the level with the same slope with increasing temperature. In case of sample #2 modeling of resistivity was possible assuming temperature-independent hole mobility equaled to experimental 1 cm^2^/Vs.

In order to further verify if the observed deep defects act as an electron or hole trap and confirm the data in a quantitative manner, Hall effect measurements at elevated temperatures were performed. It was found that in high temperatures, regime *p*-type conductivity of the investigated samples was revealed. Figure 4a,b presents the hole concentration (Figure 4a) and resistivity (Figure 4b) vs. inverse temperature for both samples. In the case of sample #2, the slope of both curves indicates the generation of holes with a conductivity activation energy of about *E_A_* = 500 meV, reflecting an approximate Fermi level position pinned at deep donor level (*E_DD_*) located at 400–500 meV above VBM. The data are derived from Equation (4), being the charge neutrality equation taking into account Mg acceptors, shallow and deep donors, and, as well as from Equation (5), making use of the changes in the occupation of the deep donors charge state. The charge neutrality is given by

(4) $$p+N_{DO}^{+}+N_{DD}^{+}+3N_{DD}^{3+}=n+N_{AMg}^{-}=\frac{N_{Mg}}{1+g_{Mg}\cdot \exp\left(\frac{E_{Mg}-E_{F}}{k_{B}T}\right)},$$

where *p* and *n* are concentrations of holes and electrons (in our case *n* is negligible, since all shallow donors act as compensating centers and do not provide electrons to the conduction band) in the valence and conduction bands, respectively, $N_{DO}^{+}—$concentration of ionized oxygen donors (equal to concentration of neutral donors *N*_DO_, since all donors compensate acceptor states), *N*_M*g*_—total concentration of Mg,$\;N_{Mg}^{-}$—concentration of ionized Mg acceptors, *g*_Mg_ is the degeneracy factor of the Mg shallow acceptor level, *E_Mg_* = 150 meV—ionization energy of Mg acceptor, *N_DD_* and $N_{DD}^{+}$*_,_* $N_{DD}^{3+}$—concentrations of neutral, singly, and triply ionized deep donors, respectively, *g_DD_*—degeneracy factor of the deep donor. In the first approximation and for the sake of simplicity, we neglect the influence of the N*_i_*–N*_i_* defect and introduce into the charge neutrality equation only multivalent N vacancies with $E_{DD}^{0/+}$ donor level and $E_{DD}^{+/3+}$ = 3000 meV below the CBM acceptor level. These N vacancies are dominant deep defects according to LPITS results and we assume they all undergo the negative-U effect. Further, we take advantage of the fact that the ratio $N_{DD}^{q-1}/N_{DD}^{q}$ between the concentrations of centers of the subsequent electron charge states *q* and *q* − 1 undergo the equation:

(5) $$\frac{N_{DD}^{q-1}}{N_{DD}^{q}}=\frac{g_{q}}{g_{q-1}}exp(\frac{E_{DD}^{\left(q/q-1\right)}-E_{F}}{k_{B}T})$$

where all degeneracy factors *g_q_*, *g_q_*_−1_ were assumed to be unity. We keep in mind that the total concentration of N vacancies $N_{DD}=N_{DD}^{0}+N_{DD}^{+}+N_{DD}^{3+}$. The combination of Equations (4) and (5) adopted for multivalent defect centers [49] is used for the description of experimental data with input parameters from SIMS or LPITS: *N_Mg_, N_DO_*, *N_DD_*, *E_Mg_*, and *E_DD_* above VBM (Figure 4). 

For this paper, a detailed analysis of the electron transport measurements was performed only for sample #2 due to a large concentration of defects in the N-sublattice. In sample #1 (treated as a reference sample with a small concentration of these defects), the defect structure is very complex. At this stage, we state that *V*_Ga_ defects (identified earlier as the T4 trap) play the most important role in its electrical properties. We see in Figure 4 that for this highly compensated sample, resistivity is much higher than in sample #2, whereas the slope of the hole concentration and resistivity corresponds to the activation energy of conductivity equal to 1.4 eV, which agrees quite well with experimentally derived *E_ADC_* = 1.7 eV (Figure 2a) and activation energy of T4 trap (Figure 3c). Thus, the Fermi level is close to the (−/2−) and (0/−) transition level of *V_Ga_* and related defects [15] (of concentration even 1 × 10^19^ cm^−3^), making presented experimental data of sample #1 consistent. The concentration of N*_i_*–N*_i_*_,_ and *V_N_* defects seems to be small (at the order of 10^17^ cm^−3^), and therefore their participation in compensation of this sample is limited. A detailed quantitative description of experimental electrical data and a discussion of the compensation mechanism are beyond the scope of this paper due to the complex defect structure of this sample. At this stage, we state that *V*_Ga_ plays a key role in explaining the experimental Hall effect data because a smaller [Mg]/[O] ratio leads to a high compensation resulting in a mid-gap position of *E_F_*, closer to the acceptor level of *V*_Ga_. In sample #2, where compensation is smaller and Mg concentration is larger, substantial total concentration of *V_N_* (8 × 10^18^ cm^−3^) and also *N_i_–N_i_* (concentration of about 2 × 10^18^ cm^−3^) are detected at LPITS measurements. In this case, the Fermi level is lower and pinned to donor levels of *V_N_* and N*_i_*–N*_i_* at about 450 meV above VBM. In the temperature-dependent Hall effect, experiment hole transition from donor levels of *V*_N_*,* mainly (3+/+), located at about *E_V_* + 0.45 eV [15], are mostly responsible for the observed transport properties. Consequently, the *p*-type conductivity at high temperatures was observed. The Hall effect data vs. temperature of sample #2 can be well described by the charge neutrality equation main parameters taken from SIMS, LPITS (*N_Mg_* = 2 × 10^19^ cm^−3^, *N_DO_* = 1 × 10^18^ cm^−3^), including the compensating deep donor concentration *N_DD_* = 1.1 × 10^19^ cm^−3^ of ionization energy *E_V_* + 450 meV, which is very close to estimated total concentration of *V_N_* and N*_i_*–N*_i_* centers (6 × 10^18^ cm^−3^). We note that some content of detected N*_i_*–N*_i_* centers (concentration 2 × 10^18^ cm^−3^) of similar ionization energy is also present. Thus, the donor defects in N-sublattice play a significant role in the compensation mechanism. Our final remark is that the presented results are consistent also from the thermodynamic point of view, as N vacancies and N*_i_*–N*_i_* are expected as compensating donors if *E*_F_ is low. 

## 4. Conclusions

The unique investigations of deep-level defects in SI AT-GaN:Mg crystals with the Mg concentrations of 6 × 10^18^ and 2 × 10^19^ cm^−3^ have been performed using the LPITS measurements and electrical transport measurements. The crystals were grown at a temperature ranging from 450 to 550 °C and a pressure of 200–400 MPa using a highly reactive supercritical ammonia solution. The oxygen concentration in both kinds of crystals was 1 × 10^18^ cm^−3^. The crystal’s resistivity was found to be dependent on the [Mg] and the lower Mg content was of the order of 10^11^ Ωcm. For the higher Mg content, the material was less compensated with the resistivity of ~1 × 10^6^ Ωcm. Both kinds of crystals were *p*-type, but the Fermi level position for the former was at around *E*_v_ + 1690 meV and for the latter at *E*_v_ + 397 meV. 

The presence of grown-in point defects formed in the crystals due to displacement of nitrogen atoms from the substitutional positions was found indirectly by comparing the activation energies of deep traps detected by the LPITS measurements with the HSE-calculated energy levels for *V*_N_ (3+/+), *V*_N_ (2+/+), and N*_i_*–N*_i_* (2+/+). These activation energies are 433, 450, and 460 meV, respectively, and were resolved using the Laplace numerical procedure based on the analysis of the photocurrent relaxation waveforms induced by the thermal emission of charge carriers. The LPITS results indicate that in the SI AT-GaN:Mg crystal with [Mg] = 6 × 10^18^ cm^−3^, the concentrations of N vacancies and N interstitials are 2.8 × 10^17^ and 3.6 × 10^17^ cm^−3^, respectively, and in the material with [Mg] = 2 × 10^19^ cm^−3^, these concentrations are by the order of magnitude higher, being 4.8 × 10^18^ and 3.6 × 10^18^ cm^−3^, respectively. The comparable values of [*V*_N_] and [N*_i_*] in both materials confirm the view that the Frenkel pairs in the N-sublattice are created during the ammonothermal growth. 

An important feature of the material with the lower Mg content is the presence of the midgap trap with the activation energy of 1870 meV attributed on the grounds of the HSE calculations to the (−/2−) transition level of Ga vacancies. The *V*_Ga_ concentration as high as ~1 × 10^19^ cm^−3^ seems to be related to the N-rich growth conditions and a light deviation of the SI AT-GaN:Mg crystal from stoichiometry. The fact that the *V*_Ga_ centers are not observed in the material with [Mg] = 2 × 10^19^ cm^−3^ presumably indicates that all the Ga vacancies in this material are occupied by Mg and there is a significant concentration of the Mg_Ga_ acceptors shifting the Fermi level closer to the valence band maximum. 

A mechanism of the Frenkel pairs formation in the N-sublattice involving the Mg concentration in the material is proposed. Because of the significantly larger Mg atomic radius compared to that of Ga, the AT GaN:Mg lattice is subjected to compression proportional to the Mg concentration. Under the compressive strain influence, the nitrogen atoms are removed from the substitutional positions, becoming interstitials. The small N atomic radius (0.071 nm) induces the fast migration of the N interstitials at the growth temperature and the creation of the N*_i_*–N*_i_* split interstitials. 

The presented results give a deeper understanding of point defect generation mechanisms occurring during crystal growth. Particularly, they show the impact of the material stoichiometry and dopants on the properties and concentrations of native defects in crystalline materials exemplified by AT-GaN:Mg. In this aspect, the results of this work can be used to optimize the growth conditions in terms of obtaining the crystals with tailored properties, required for specific applications. Additionally, the results demonstrate a new methodological approach to the investigation of point defects in crystalline materials. It is shown that the defect properties can be analyzed on the grounds of the results of advanced calculations based on density functional theory compared with the results of experiments allowing for the determination of the defect energy levels with a sufficiently high resolution.

## Figures and Tables

**Table 1:** Comparison of resistivities and determined by SIMS concentrations of Mg, O, and H in the samples #1 and #2 of SI AT-GaN:Mg crystals with various Mg content.

Sample	[Mg] (cm^−3^)	[O] (cm^−3^)	[H] (cm^−3^)	ρ at RT (Ω cm)
#1	6 × 10^18^	1 × 10^18^	3 × 10^18^	~1 × 10^11^
#2	2 × 10^19^	1 × 10^18^	7 × 10^18^	~1 × 10^6^

**Table 2:** Activation energies *E*_a_, pre-exponential factors *A* and concentrations for deep traps detected by LPITS in samples of SI AT-GaN:Mg crystals with various Mg concentration.

Trap Label	*E*_a_ [meV]	*A* [K^−2^s^−1^]	Trap Concentration [cm^−3^]	
			[Mg] = 6 × 10^18^ cm^−3^	[Mg] = 2 × 10^19^ cm^−3^
T1	433 ± 5	1.8 × 10^4^	9 × 10^16^	8.2 × 10^18^
T2	450 ± 7	1.0 × 10^5^	2.8 × 10^17^	4.0 × 10^18^
T3	460 ± 8	7.8 × 10^5^	1.8 × 10^17^	1.8 × 10^18^
T4	1870 ± 10	1.7 × 10^9^	~1 × 10^19^	not detected

**Table 3:** Tentative identification of the deep traps resolved by LPITS in SI samples AT-GaN:Mg through comparing the trap activation energies with the energies for the charge state change in native defects calculated by using HSE hybrid functional [15,16].

Trap Label	Activation Energy from LPITS [meV]	Energy for the Charge State Change [meV] [15,16]	Trap Identification	Charge State Change	Capture Cross-Section for Holes (*σ*_p_) [cm^2^]	Defect Type
T1	433	450–540	hole trap related to nitrogen vacancy *V*_N_ (3+/+)	(3+/+) due to 2 holes emission	*σ*_p_ = 2.84 × 10^−18^	Donor, negative U
T2	450	470	hole trap related to nitrogen vacancy *V*_N_ (2+/+)	(2+/+) due to hole emission	*σ*_p_ = 1.58 × 10^−17^	Donor
T3	460	500–510	hole trap related to split interstitial N*_i_*–N*_i_* (2+/+)	(2+/+) due to hole emission	*σ*_p_ = 1.23 × 10^−16^	Donor
T4	1870	1870	hole trap related to gallium vacancy *V*_Ga_ (−/2−)	(−/2−) due to hole emission	*σ*_p_ = 2.70 × 10^−13^	Acceptor

## Data Availability

The source data presented in this study are publicly available at https://doi.org/10.18150/LLRRQS.
